# Hospital Fittings

**Published:** 1898-04-02

**Authors:** 


### The Institutional Workshop.

### HOSPITAL FITTINGS.

### A YEAR'S IMPROVEMENTS AND NOVELTIES.

Brompton Hospital for Consumption.

Here an excellent full-size **Billiard Table** for the use of the patients is a useful acquisition, and before very long it is hoped the nurses' home, so long required, will be in existence. The old houses now occupying the future site are being rapidly demolished.

Cancer Hospital, Brompton.

This hospital is beautified by numerous engravings of masterpieces; some of them signed proofs, the gifts of friends. In the bow windows of the wards seats have been placed; and hanging thermometers of porcelain have replaced those of less strictly hygienic make. At this hospital the handsome **Operating Theatre**, with all its modern fittings, is a recent addition.

Evelina Hospital for Children.

Miss Getz, the ward superintendent, has had a number of ideas of her special devising carried out at her own expense, and has presented them to the hospital. One of the most recent additions to the ward fittings is an **admirable Test Case**, with polished wood framework and glass shelves, one side for the tubes, while on the other is a securely-locked cupboard for the acids which are contained in amber-coloured glass bottles. The cases have been made from Miss Getz's design by Mr. Sexton, Southwark Bridge Road.

A simple **Plan for Keeping Enema Syringes** in good order may be seen in the ward sculleries, where a wooden bracket, something like a pipe rack, keeps them suspended above the sink when not in use.

One of Messrs. **Down Bros'. Folding Operation Tables** is a recent addition to one of the wards, where, in the event of an operation taking place in the ward, it is found extremely convenient. When not required it packs away into a small space.

Miss Getz has also added to the hospital appliances an **"X" Ray Apparatus**, the **"Carrier"** for which has been specially designed by Mr. H. J. Wagg, 15, Great George Street, consulting electrical engineer to the hospital.

The table is designed to contain all the apparatus on the lower part, while the patient lies on the table. When not in use the table is like a log on castors, the height of an ordinary table, and about 20 inches broad by 30 inches long, the top being made of some wood very transparent to the rays, such as American white wood, teak, or pine. The front is made of glass, and the ends, when unbolted and raised, form an extension to the table, making it in all about six feet long. The back consists of a pair of doors which can be locked, preventing anyone from using the apparatus who does not possess the key. The tube is mounted on a carrier, which can be moved to any part of the lower side of the table-top. The tube is worked by a 6-inch spark coil (although a 10-inch would be more satisfactory), and the coil derives current from a set of accumulators which travel on castors underneath the body of the table, so that they may be removed without any difficulty for recharging, but they are so arranged that when the table is moved they always follow, unless the table is lifted. The coil is switched on by inserting a plug in the table-top. To maintain a high vacuum in the tube a 32 C.P. lamp is kept burning below it, this being carried on the same carrier as the tube. There is also an arrangement of parallel bars fixed on the top of the table for photographic plates, or a fluorescent screen; by means of thumb screws these may be adjusted in any position.

East London Hospital for Children, Shadwell.

Here great economy in **Lighting** has been effected. The illuminant is gas, and the apparatus of the Gas Economising Improved Light Syndicate (Rochester Buildings, Leadenhall Street) has been adopted and fitted by them free of charge. The company's remuneration is half the saving effected by the use of the apparatus. The principle by which the saving is effected is as follows: Ordinary gas is passed through a screen formed by a number of wicks kept automatically saturated with carburetted fluid. The gas thus treated gives a bright and white light. It produces less heat, and half the quantity only is required to secure an equal amount of illumination. The hospital managers calculate that £60 per annum is saved by them on their gas bill. The intended convalescent home for the hospital is advancing satisfactorily towards completion.

Guy's Hospital.

"Bright's," the "pay" ward at Guy's, has lately been supplied with **new Bedsteads**, planned and designed by Dr. Perry and Miss Nott-Bower. They have several special points. In the first place the centre part of the bed-head, formed of upright bars which afford no lurking places for dust, is so made as to slide up and down the corner posts. This bed-head, when the lower end is pulled forwards makes a first-rate back-rest. If it is necessary to give an anæsthetic in the ward this centre-piece can be dropped down or taken off, leaving the head of the bed clear. Another advantage to be noted is the arrangement by which the wire springs are fastened severally into the iron frame of the bedstead itself, so that each can be easily tightened as required. The foot-rail is level with the top of the mattress. These bedsteads are made for the hospital by the Bermondsey Bedstead Company.

The electric light has been put into these wards recently, a switch being placed at the head of each bed.

A good idea has been carried out in the way of a **Stand for Macintoshes** in the wards. This consists of a polished wood frame-work fitted with brass swinging rails over which the macintoshes are hung, a division down the middle keeping bed and dressing macintoshes apart. Turkey red curtains are drawn along both sides, and the top of the stand makes an excellent place for pots of flowers or other ward accessories.

**A new Locker** also claims attention. This is made with a cupboard below and two shelves above, the idea being to keep the various belongings of each patient as separate as possible, the top shelf being a moveable enamelled tray, which may be used for dressings and then taken bodily away for cleansing purposes.

In the quarters fitted up for the additional nurses who have been added to the staff during the past year, a **good Wardrobe and Dressing-table combined** is allotted to each cubicle. It is made of polished pine. One side is a long dress cupboard, with a small shelf above the hanging-hooks; the other is a dressing-table, with swing glass, two little drawers and four larger ones, the whole width at the bottom being occupied by a long drawer. The Standard Folding Bed Company are the makers of this furniture.

Great Northern Central Hospital.

At this hospital further accommodation is again about to be opened for the use of patients, and one of the unoccupied wards built and furnished four years ago will be speedily in use.

Grosvenor Hospital for Women and Children.

The Grosvenor Hospital has entered on a new existence during the past twelve months, the present building, of which plans were published in The Hospital for November 20th of last year, only being opened for occupation in the autumn. The **Ward Furnishing** has been carried out by Messrs. Heal, Tottenham Court Road, and all the fittings are good, and up to date. The beds stand higher than usual, for convenience in nursing. The lockers beside each bed have tiled tops, and a small polished wood box stands under each bed, these being for the reception of the patients' belongings. In the theatre, the fittings of which were done by Messrs. Dove Bros., Tokenhouse Yard, the walls are lined with white tiles; the sinks, &c., are Messrs. Dent and Hellyer's, the water taps for the hand-basins being worked by foot-pressure; and the stands and tables, of glass and metal, are supplied by Messrs. Arnold and Son. Antiseptic solutions are kept in a swinging "bottle-stand."

Hospital for Sick Children, Great Ormond Street.

A magnificent **Polyphone** has been presented to this hospital, which gives the small patients a great deal of pleasure, and adds to the funds of the hospital by a penny-in-the-slot arrangement, by means of which visitors to the wards can set the instrument in motion.

Hospital for Diseases of the Chest, Victoria Park.

This beautiful and most needed hospital has been enabled to reopen a number of beds, raising the provision for patients from 16 to 65 beds.

King's College Hospital.

Amongst "new appliances" at King's College Hospital may fairly be reckoned the recently-fitted **Ophthalmic Operating Theatre**, with all its up-to-date appurtenances. It is curiously irregular in shape, lighted by a very large window formed of a single sheet of plate-glass. Walls and ceilings are lined with opaline tiles, white, except where the corners are picked out with a border of dark red; the floor is laid with terrazzo, and its corners rounded to avoid dust particles. Warmed and purified air is driven through the theatre by means of an electric fan, the place itself being heated by hot coils running behind or, rather, through the walls. They are cleverly utilised in one place, where slate shelves, embedded in prepared sterilised wool, are arranged over them, enclosed in glass panels. These are for the reception of instruments or dressings, and for the large glass bottles containing carbolic and other antiseptic lotions, all of which are thus kept at the proper temperature for use when the theatre is heated for operations. Patients are brought into the theatre on their beds, so that no table finds a place in the eye theatre. All instrument and dressing stands are of enamelled iron, with glass trays. The whole theatre, with its fittings, is the gift of the family of the late Sir George Johnson, physician to the hospital, a brass plate on the door setting forth this fact. The work has been carried out under the directions of Professor McHardy.

In the wards there are several novelties to notice. Miss Monk has an inventive brain, which is ever at work for the good of her hospital, and the results of her plannings are always to be chronicled with admiration. Quite lately a delightful "**Test Case**" has made its appearance on one of the ward tables, designed by Miss Monk, and made for her by Messrs. Maw, Son, and Thompson. The case is of polished wood, large enough to contain twelve bottles, with a hinged front like a writing-case that shuts down and locks. The special feature to be noticed is the long slit at the back to allow of free ventilation, and prevent the accumulation of noxious fumes such as will arise from strong acids enclosed in an air-tight case. The bottles fit into trays, which are removeable, and so can be easily cleaned. There is a small drawer at the bottom of the case. Ranged beside the test case are trays, divided by lines into squares numbered to correspond with the beds. On these are placed the test tubes, and to still further obviate any chance of mismatching a tiny zinc label belongs to each tube, also numbered. These trays are the invention of Sister Kate.

**An Admirable Ward Table** has been introduced into one of the wards, also designed by Miss Monk. It is oblong in shape, one entire side being fitted with drawers devoted to surgical dressings and appliances, and a cupboard for basins, trays, &c. The other side is arranged with a centre knee-hole to form the sister's writing-table, the drawers on either side containing the ward stationery. The whole is made in polished pine; doctors' basins, flower pots, and other things being arranged on it. The table stands in the centre of the ward. Another table is for dressings only, the top being a large slab of ground glass on galvanised iron supports. A wooden shelf below holds instrument cases, &c. Two towel rails are fixed at either end, and along one side runs a brass rail to protect the big ward bottles of carbolic, perchloride, and other antiseptics which stand upon it from being accidentally knocked over. These tables are made by the hospital carpenter from Miss Monk's designs.

Very nice **New Bedsteads** have been supplied throughout the hospital. They are of dark painted iron, with very low head rails, for convenience in the event of an anæsthetic being given in the ward, and the springs have been arranged on a level with the side rail to admit of fracture boards being placed on them without difficulty. In the ophthalmic wards the bedsteads are made with a removeable head rail for convenience when in the theatre, patients from these wards being taken straight to the theatre on their beds. The bedsteads are made for the hospital by the Longford Company.

### London Hospital.

Some **New Beds** of improved height have been added to the wards, and the bed-table at use at the Poplar Hospital has also been adopted here. A **Macintosh Sheet airer** has been found most satisfactory; it is in the form of an upright case. The advantages of this case in which the macintoshes are hung are its convenience, and the fact that the macintoshes thus preserved last twice as long as when kept in other ways. Some of the wards have been covered with indurated linoleum, which has a good effect, and is reported to be satisfactory in other respects. This floor-cloth was presented to the hospital, and was supplied by Messrs. Treloar.

### London Temperance Hospital.

So many good things have fallen to the share of the Temperance Hospital of late that the difficulty is to explain them within the required space. In the first place, one of the male adult wards has been **tiled throughout with opalite**, neutral tinted, and the effect is altogether charming. Just inside the door is a new opalite slab for testing purposes, with an aluminium stand for tubes. A dressing-stand of opalite and metal, and a stand for Leiter's tubes, of plain glass with a brass frame, are in this same ward.

In the **"Aseptic" Ward**, for one bed only, which, it will be remembered, was opened in January, everything is of the most approved modern fashion. The walls and ceilings are lined with opalite tiles, the floor is of marble mosaic. Air, filtered through cotton wool beds, is driven into the room by an electric fan. There is a Pridgin Teale fireplace. The locker is of aluminium, with opalite top; even the chair is of metal, and all tables and dressing-stands are of metal and glass. Even the frame of the chintz screen is of this material. The syringes and other appliances are made of aluminium, which has the great advantage of requiring little or no polishing. These various novelties have been supplied by different firms, some of the tables and stands coming from Messrs. Maple, while other appliances have been made by Messrs. Maw, Son, and Thompson. The tiling in the wards and in the corridor outside, as well as in the small bath-room especially attached to the new ward, is the work of the Opalite Company, Bishopsgate Street Without. The sluices there and in the bath-room of the children's ward, which are a new addition, are Doulton's patent.

### National Hospital for Paralysed and Epileptic (Albany Memorial), Queen-square.

**The Children's Ward** at the National Hospital has been supplied with **new cots**. These are made in enamelled iron, with wire springs, and sliding side rails, the latter when in position being held in place by a support along the side, out of reach by mischievous fingers. Some of the cots are fitted with sockets at the corners, into which can be easily fixed the frame of a steam tent. They are all made somewhat higher than ordinary children's cots in order that the massage, which is so important a feature of the treatment at this hospital, may be more easily carried out.

Almost every ward has been provided with **new bed-boards** of a very good pattern. They are made of dark polished wood, with narrow hinged flaps on both sides to keep in position, on one, the patient's card, on the other, the prescription paper, and are completed by a brass hook by which to hang them upon the end of the bed. They are made for the hospital by Messrs. Cross.

### Poplar Hospital for Accidents.

At this hospital improvements in the lighting have been effected by the use of **Incandescent Burners.** These have been supplied in pale pink opal glass shades which render the light a very pleasant one. Dark green shades have also been supplied in some cases to further modify the light. A new **Operating Theatre** has been added with terrazzo floors, polished walls with rounded corners, and fitted with glass and metal furniture. The ceiling is of enamelled iron, and Messrs. Down's excellent sinks with foot treadles for the control of the water supply have been adopted with a special mechanical appliance for a continuous supply of water at will when the foot is removed from off the treadle.

The **New Beds** supplied to this hospital are slightly higher than those before in use, for the purpose of causing less fatigue to the attendant nurses. Very nice **Bed-tables**, made by the joiner specially employed by the hospital, have been recently introduced, consisting of a slender wooden tray wide enough to reach across the bed and supported on four legs. The isolation block and the out-patient department are new, as is also the box-room situated under the out-patient department. These buildings are heated throughout with hot water. The walls in one ward are covered with enamelled iron casing.

### Royal Free Hospital.

The Royal Free Hospital is able to boast of several acquisitions during the past year. One, a large **"Down's" steriliser** for dressings, is a gift from Miss Wedgwood for the use of the theatre, where its possession has proved a comfort to all concerned. A glass and enamel table has also been added to the theatre appliances, this, too, from Messrs. Down Bros., as well as a delightful little contrivance for boiling needles in the shape of a nickel-plated basket with perforated bottom, like a sugar sifter, wherein the needles are placed before being plunged into the steriliser. Another little rack is to place scalpels in for the same purpose.

In the wards, glass slabs have been added to ward tables; and in one ward a new test-table, wood, with glass shelf, has been presented by the friends of a patient. The cupboard, wherein stand the testing acids, is on the wall behind the table. A similar test-table has been given to another ward by one of the medical staff. Each ward is the richer, too, for a couch, an easy or wheel chair, provided by the Samaritan Fund of the hospital for the convalescent patients, and also for three new bed-tables. All these things have been supplied by Messrs. Atkinson, Westminster Bridge Road. A very nice **screen**, fourfold, of dark polished wood frame, and turkey-red curtain, has been presented to one of the wards, and in the same one two Pridgin Teale grates have lately been put in, looking very well indeed with their dark red tiles and generally cheerful appearance.

### St. Bartholomew's Hospital.

At this hospital experiments are in progress with a **New Stove.** It is encased in Doulton tiles of a rich brown, and stands well forward in the ward. A cheerful open fire adds to its advantages. The outside air is conducted to the stove by means of a flue running under the floor, and, after passing over hot pipes, enters the ward through the ventilating grating, which forms an ornamentation of the stove. There are, however, artistic drawbacks in the appearance of the stove. Hot-water radiators (Cannon's Patent Radiating Coils) have also been added to the wards of this hospital, and are much approved of, as it is found that by their means the temperature can be easily regulated.

The **New Beds** in the wards of this hospital are made higher than formerly, and are supplied with large-mesh copper wire netting, with a row of strong springs beneath this at the top and bottom of the bed: they are considered firm enough for the use of fracture cases. Some **New Lockers** are made of dark oak, and are adapted to form low comfortable chairs by the side of the bed. Below the seat is a receptacle for clothing, and the back can be adapted to form a bed-table. A recess also in the back gives a place for books and papers. The makers of these lockers are Messrs. Beer and Cash, 13, Warfe Road, City Road; and they are known as Ovenden's Patent.

St. George's Hospital.

A very desirable addition has been made to the **Mortuary** arrangements at St. George's Hospital during the past year. This is a small "chamber," next door to the mortuary proper, where after a death in the wards the shell is taken for the friends to visit. Here they find their dead lying peacefully amidst reverent and Christian surroundings, the aspect of the little chapel bearing testimony to the fact that fitting care is taken of the earthly remains of those who die in the hospital. The tiled walls are decorated with sacred designs, and texts; the floor is of terrazzo. A marble slab carries vases of fresh flowers. Thorough ventilation is effected by top cross-lights in the roof. The decoration has been carried out by Messrs. Simpson and Co., Long Acre, under the supervision of the chaplain, who has taken much interest in initiating this reform. Far too little heed is taken in the matter of reverently caring for the dead at most institutions, and no little violence is sometimes done to the feelings of sorrowing friends, so that this is a welcome improvement to chronicle at St. George's. The mortuary itself has also been throughout lined with white tiles, and all its arrangements are excellent.

Another much-needed improvement it is pleasant to record is that of the provision at last of **a Proper Office for the Matron.** This is a pleasant, good-sized room, on the ground floor, with the advantage of a convenient entrance lobby. We remember not so long ago having to stand in the basement, hustled by porters, tumbling over coal-boxes, and other impedimenta, in dismal darkness, waiting for admittance to the matron's office, then next door, if we are not mistaken, to the porter's room, the said office being certainly itself not arge enough to swing therein the proverbial cat. St. George's and the matron are to be congratulated on now possessing an office worthy of one of our largest hospitals.

St. Mary's Hospital.

**New Hot water Radiators,** from Messrs. Haddon and Son, have been adopted in the new wing of this institution. The basement of the new wing is now nearly ready for occupation. The building promises to be a handsome one. The out-patients' hall is supported by massive pillars faced with Doulton tiles, some bearing a Royal crown over a large C. The floor is of teak. The building is to be lighted by electricity. In some of the old wards some charming mural paintings have been introduced, and in "Thistlethwaite" the Sister has placed a carved oak chest for the use of theatre appurtenances. In "Forester's" Ward a musical instrument—a **Polyphone**—has been presented by Captain Travers for the use of the patients. Some **New Beds** have been introduced into the wards. Their particular feature is the employment of broad laths of zinc in place of the narrow iron ones which are usually employed. These laths are much approved, as they give a firm bed suitable for fractures, and do not cut the hair mattrass.

St. Thomas's Hospital.

In the wards of this hospital there are several novelties to be noted. The **Ward Tables** have been fitted with glass tops, and **new Dressing Wagons** supplied. These latter have also glass and tiled shelves, with tin-lined drawers for dressings, &c., on one side of the top shelf being fixed a support for the large bottles of antiseptic solutions, from which the supply is drawn by taps, underneath being fixed a narrow zinc tray to catch stray drops. The table moves easily on castors. The makers are Messrs. Mathews, New Oxford Street.

Very nice new **"Bentwood" Couches** have also been added to the ward furniture.

Other alterations are in course of arrangement in some of the wards, where the very large kitchens are being divided up, one part to become a linen-room as in the newer wards. The partitions here, and those in the new home, are of metal, "Potter's Patent Expanded Metal Partitions" (39, Victoria Street).

The Middlesex Hospital.

For some little time past **an "X" Ray Photographic Department** has been in working order at the Middlesex Hospital, and has proved very useful. To convey the apparatus from ward to ward, and wherever required, a convenient "carrier" has been designed by Mr. C. H. March (secretary's clerk), and made by the hospital carpenter. A more recent development is the establishment of an ordinary photographic department, certain to be of great value in many ways.

The West London Hospital.

The West London Hospital is preparing to open one of its **New Wards,** and accommodation for the nurses, which is badly needed, is being provided in a house which belongs to the hospital.

Westminster Hospital.

A **New Operating Theatre** has been added to the Westminster Hospital. The students' gallery in this building consists of a slab, underneath which are brass gratings for the admission of warm filtered air. The windows in the theatre are all plate glass framed in enamelled iron. The drainage is carried through into the open before being conducted to the pipes leading to the sewer, in order to prevent any possibility of sewer gas finding its way in any circumstances whatever into the theatre.

